# Bone Mass and Strength and Fall-Related Fractures in Older Age

**DOI:** 10.1155/2019/5134690

**Published:** 2019-09-09

**Authors:** Kirsti Uusi-Rasi, Saija Karinkanta, Kari Tokola, Pekka Kannus, Harri Sievänen

**Affiliations:** ^1^The UKK Institute for Health Promotion Research, Tampere, Finland; ^2^Department of Orthopaedics and Traumatology, Tampere University Hospital and Medical School, University of Tampere, Tampere, Finland

## Abstract

**Introduction:**

Low bone mineral density is a risk factor for fractures. The aim of this follow-up study was to assess the association of various bone properties with fall-related fractures.

**Materials and Methods:**

187 healthy women aged 55 to 83 years at baseline who were either physically active or inactive were followed for 20 years. They were divided into two groups by whether or not they sustained fall-related fractures: fracture group (F) and nonfracture group (NF). At baseline, several bone properties were measured with DXA and pQCT, and their physical performance was also assessed.

**Results:**

During the follow-up, 120 women had no fall-related fractures, while 67 (38%) sustained at least one fall with fracture. NF group had about 4 to 11% greater BMD at the femoral neck and distal radius; the mean differences (95% CI) were 4.5 (0.3 to 8.6) % and 11.1 (6.3 to 16.1) %, respectively. NF group also had stronger bone structure at the tibia, the mean difference in BMC at the distal tibia was 6.0 (2.2 to 9.7) %, and at the tibial shaft 3.6 (0.4 to 6.8) %. However, there was no mean difference in physical performance.

**Conclusions:**

Low bone properties contribute to the risk of fracture if a person falls. Therefore, in the prevention of fragility fractures, it is essential to focus on improving bone mass, density, and strength during the lifetime. Reduction of falls by improving physical performance, balance, mobility, and muscle power is equally important.

## 1. Introduction

Fragility fractures are associated with increased morbidity and mortality, and low bone mineral density (BMD) is one of the factors influencing the risk of fall-related fractures among older women. Several factors influence bone health, not only lifestyle habits including nutrition, physical activity, and smoking but also many diseases and drugs. Since most fractures are caused by a fall, falls reduction is the key component in fracture prevention. Declined physical performance or balance predisposes to falls as well [1–3].

Weight bearing physical activity is essential for normal development and maintenance of a healthy skeleton. It is also essential for good physical fitness and functioning. Both strong bones and good physical functioning are, in turn, associated with lower incidence of injurious falls and fractures [4]. In their review, Howe et al. suggested that a relatively small but possibly important effect of exercise on BMD can be seen in postmenopausal women [5]. In a long-term exercise study with a multipurpose program, Kemmler et al. showed that BMD declined in both exercise and control groups, but the decline was smaller in the exercisers [6].

Clinical prevention of fragility fractures is largely based on the ability to estimate fracture probability by means of risk factor assessment, such as FRAX [7]. However, BMD measured by DXA alone or FRAX (with or without DXA) cannot identify accurately individuals who will sustain a fracture [8]. This is simply because many fractures, particularly in older populations, are a result of a fall, which are, in turn, influenced by several environmental and other medical causes (e.g., impaired visual function, muscle strength, and balance) [9].

Besides low BMD, several other bone properties, such as bone size, geometry, cortical thickness, and area as well as trabecular bone density are important risk factors for fractures. The most commonly measured clinical bone property, DXA-based areal BMD, represents an integral of contribution of both cortical and trabecular bone sizes and densities within the scanned volume [10]. It therefore lacks the ability to measure properties that contribute more specifically to the ability of bone to withstand loads caused by a fall. With peripheral quantitative computed tomography (pQCT), cortical and trabecular bone properties can be evaluated [11].

In this 20-year register-based follow-up of older physically active or sedentary postmenopausal women, we evaluated the relationship between several bone properties measured with DXA and pQCT at baseline and incident bone fractures.

## 2. Participants and Methods

### 2.1. Participants

At baseline, all participants were either physically active or sedentary postmenopausal nonsmoking women aged from 55 to 83 years. Physically active women were recruited from local gymnastic clubs, and they had engaged in recreational gymnastics or folk dance at least twice a week for more than 20 years. Sedentary controls were recruited via a local newspaper advertisement, and they had no more than once a week light to moderate exercise which was not gymnastics [11].

From the original cohort of 243 women, 187 (77%, 103 exercisers and 84 controls) were included in this register-based follow-up study. We had no access to data of 56 women (49 were living outside the city of Tampere, and for seven women the only existing information was death). Fallers with fractures (F) and those without fall-induced fractures (NF) during the 20-year follow-up period (September 1997–April 2018) were evaluated from medical records. The follow-up time was calculated from the baseline measurements until the end of April 2018 or until the date the participant had moved from the area or died.

The study protocol was approved by the Human Ethics Committee of the Tampere Region (approval 53/2017). The use of the patient-register data was further approved by the Department of Social Services and Health Care of the city of Tampere.

### 2.2. Methods

Physical performance and bone measurements were done at baseline. Fractures were evaluated during the 20 years' period from the baseline onwards.

Areal BMD (g/cm^2^) of the femoral neck on the dominant side, the femoral trochanter, and the distal radius were measured with dual-energy X-ray absorptiometry (DXA) (Norland XR-26, Norland Corp., Fort Atkinson, WI) [11].

In addition, the tibia was evaluated with peripheral quantitative computed tomography (pQCT) (Stratec XCT 3000, Pforzheim, Germany). The tomographic slices were taken from the midshaft and distal part of the right tibia. For the tibial shaft, the BMC, cortical cross-sectional area (CoA, mm^2^), cortical density (CoD, g/cm^3^), and density weighed section modulus (BSI, mm^3^) were determined. For the distal tibia, the evaluated parameters were BMC, total cross-sectional area (ToA), trabecular density (TrD, g/cm^3^), and BSI [11].

The maximal isometric strength of the leg extensors was measured by a strain gauge dynamometer, and the maximal grip strength of the dominant forearm was determined with a standard grip strength meter. Leg-extensor power was evaluated with a vertical countermovement jumping test, using a contact platform (Newtest, Oulu, Finland) and recording the flying time of the jump. The height of the jump (*h*) was calculated from the flying time as follows: *h* = *gt*^2^/8, where *g* is the 9.81 m/s^2^ and *t* is the flying time in seconds. Dynamic balance was tested by a figure-of-8 running test, the test being performed by running around two poles placed 10 m apart. Cardiorespiratory fitness (estimated maximal oxygen uptake, VO_2max_) was assessed by a standardized 2-kilometer walking test [11].

Fall-related fractures of the participants were scrutinized from a computerized patient register from September 1997 onwards (Pegasos patient information system, CGI, Finland) in the city of Tampere. A fall was defined as “an unexpected event in which the participant comes to rest on the ground, floor, or lower level” [12]. Only falls that caused a fracture were included. A faller with fracture (FF) was a person who had at least once contacted the healthcare system due to a fall with a consequence of fracture during the 20-year follow-up period. Those women who had either contacted the health care due to a fall-related injury other than a fracture or had no fall-related contacts to health care were considered nonfracture (NF) cases. The years between 1997 and 2002 were manually examined from paper files, while more recent patient data were accessed in the digitalized form.

### 2.3. Statistical Analysis

The participants (*n* = 187) were divided into two groups by whether or not they had broken their bones as a result of a fall, i.e., fracture (F) and nonfracture (NF) groups. Differences in bone properties and physical performance between the F and NF groups were evaluated with analysis of covariance (Ancova). Analyses were adjusted for baseline age, height, and weight as possible confounders.

In addition to the above primary-analysis, the age, height, and weight-adjusted mean differences between F and NF groups were evaluated separately in the baseline exercisers and sedentary control groups.

The participants were also divided into two groups by median of femoral neck BMD. The Cox proportional hazard regression model was used to calculate hazard ratio (HR) for lower vs. higher BMD-groups. Also, this analysis was adjusted for baseline age, height, and weight.

SPSS 25 statistical software (IBM Corp. Released 2017. IBM Statistics for Windows, Version 25.0. Armonk, NY: IBM Corp.) was used for all statistical analyses. *P* values were 2-sided and those less than 0.05 were considered statistically significant.

## 3. Results

The baseline characteristics of the participants are given in Table 1. During the 20-year follow-up, 67 (38%) out of 187 women sustained at least one fall that caused a fracture. The total number of fractures was 113. The most common fractures were upper limb fractures (total 48) followed by 12 hip or pelvic fractures, 10 vertebral, and 9 lower limb fractures. At baseline, there were no statistically significant differences in anthropometry or age between F and NF groups.

**Figure 1:**
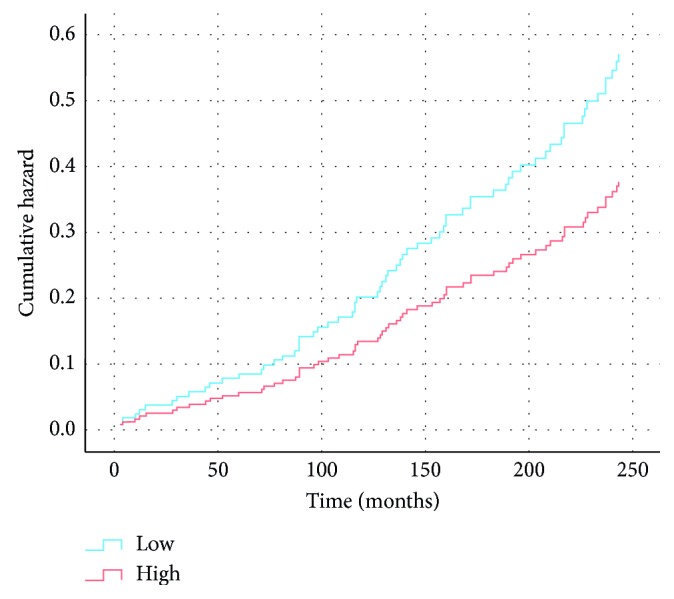
Hazard ratio for fracture fallers in low vs. high BMD-groups based on median BMD of the femoral neck at baseline, adjusted for age, height, and weight.

At baseline, F group had lower DXA-based BMD at the distal radius and femur, the mean differences ranging between 4 to 11%. The greatest mean difference (95% CI) was at the distal radius 11.1 (6.3 to 16.1) %. The corresponding mean difference at the femoral neck was 4.5 (0.3 to 8.6) %. Also, bone properties were lower at the tibia except for CoD and BSI of the tibial shaft. There were no statistically significant mean differences in physical performance (Table 1). Between the lower and higher BMD-groups, HR (95% CI) for fracture was 1.52 (0.89 to 2.59) greater in the lower BMD-group compared with the higher BMD-group (*P*=0.13). Cumulative hazard in these groups is shown in Figure 1.

In both exercisers and controls, roughly one-third (37% of exercisers and 35% of controls) were fracture fallers. When comparing the exercisers and controls with and without fractures within the groups, all absolute bone property values were higher in NF groups, but adjusted mean differences were statistically significant only at distal radius BMD and distal tibia BMC. There was a trend for better physical fitness among the exercisers, favoring F group (Table 2), but only time difference in the figure-of-eight-run test (agility) reached statistical significance. In the control group, there were no differences in physical fitness between the F and NF groups (Table 2).

## 4. Discussion

Our results showed that the baseline areal BMDs both at the distal radius and proximal femur were lower in fallers with fractures than those without. Also, women who sustained a fracture because of a fall had lower trabecular BMD, smaller cortical area, and weaker bone structure at the tibia, evident as a significantly lower BMC. In contrast, there were no differences in physical performance.

When considering the participants in the original groups, the exercisers had somewhat greater tibial BMC and cortical area at the tibial shaft and significantly better physical performance than the sedentary controls. Further, in both exercisers and controls, women who sustained a fall-related fracture had somewhat lower absolute bone mass and strength, although the adjusted mean differences were statistically significant only at distal radius BMD and distal tibia BMC. The only difference in physical performance was in agility in the exercisers favoring the women without fractures.

The most common type of fracture in the present study was an upper limb fracture. Wrist fractures are more common than other fall-related fractures, and its incidence starts to increase at younger age [13, 14]. Moreover, wrist fractures often represent a seminal event in patients at high risk for later hip fracture. Recurrent falls and impaired balance are among the most important risk factors for falls [15, 16].

Bone is dynamically remodeled throughout a person's life. While bone strength is proportional to the square of its volumetric BMD [10] especially at the lower limbs, small decreases in bone density are partly compensated by increased bone size. Eventually, the cortex may become too thin and weak and is more likely to break due to a minor injury, such as a low-energy fall [17].

The effects of physical activity or exercise on bone are highly site-specific, and mainly forceful, rapid movements or impacts that load the bones from many directions are most beneficial [18]. However, with ageing, high impact activity may diminish due to limited physical capacity or comorbidities. Among older adults, a large number of repetitive movements or low-impact exercise with moderate power and speed is probably more realistic and achievable. In the present study, the main type of physical activity of the exercisers was low-impact recreational gymnastics, which was done regularly (at least twice a week) for several decades (the mean duration at baseline was over 30 years).

The incidence of fractures is substantially lower than the incidence of falls; about 10% of falls result in fractures, which emphasizes the role of fall prevention in preventing fractures. Risk factors for fractures can be classified into factors that are related to bone properties and factors related to falls [19]. Although exercise has the potential to avert bone loss in older age [5], more important is its role in improving physical functioning, balance, and mobility and thus decreasing the risk of falls. Whereas physical activity has not always proved effective in reducing falls [20, 21], exercise training may help to prevent fall-related injuries [22–24].

Declined physical fitness and functioning have been shown to be associated with increased risk of falls and fractures [25, 26], but in this study there were no between-group differences in physical fitness between the F and NF groups. This may be due to rather even proportion of exercisers and sedentary controls within these groups. When comparing exercisers with sedentary controls, the analyses showed a clear benefit in physical performance favoring the exercisers. Among exercisers the mean difference in agility was statistically significantly better in the NF group than F group. Although it is likely that physically active life style is maintained as long as possible, it is very likely that the amount or intensity of physical activity declines with age [27]. Apparently, this concerns even healthy older adult who have previously been regularly physically active. Our study groups are well comparable to the groups in a long-term study by Kemmler et al. [6]. In that study, women were allowed to select between the exercise group and the control group, and previously active women were more likely to select the exercise group. Apparently they would have been more physically active also without the given exercise program, similar to the exercisers in this present cohort [28].

One of the strengths of our study is a long follow-up time of the cohort. In addition, fractures were acquired from medical records filled by healthcare professional during and after the patients visits and confirmed by related radiographic reports. A limitation is that we had no information about physical activity or physical performance towards the end of the follow-up. However, physical activity was well evaluated at baseline, and the exercisers were more fit than physically inactive controls suggesting that the difference in leisure physical activity was real [11]. Six years later, in spite of slightly decreased physical performance in both exercisers and controls, the mean differences in physical performance had been maintained [28]. Mobility disability is a dynamic process, and it is probable that the motor functions and muscle strength decline in time due to ageing and comorbidities. Moreover, diseases or drugs may affect risk of falls, and even temporary difficulties in mobility may predispose to falls [29]. Unfortunately, we had admission only to fall-related healthcare visits and no access to medication or other health data.

We also had no information about falls that did not require healthcare services, and it is not known if the F group had more falls in general than NF group or whether they were just more prone to sustain a fracture when falling. However, these groups with and without fall-related fractures differed significantly at baseline suggesting that declined bone properties at baseline made these women more likely to sustain fractures when falling. Although diagnosis of osteoporosis was not common at baseline (only 25 women, 13% had osteoporosis), the proportion of those with osteoporosis might have increased in time. It is noted that that the study groups were not likely representative of general female population at given age but rather that of a healthier fraction at baseline.

The study was underpowered for subgroup analyses of the bone data, and despite the trend favoring the NF group, mean differences did not reach statistical significance between F and NF groups within the exercise and control groups. However, the trend in both exercisers and controls give support to the finding of the primary analyses; declined bone properties predispose to fractures when falling despite physical performance.

## 5. Conclusions

Although there are several risk factors affecting the risk of fragility fractures, low bone density, mass, and strength contribute to higher fracture risk if falling. In fracture prevention, it is essential to improve both bone health and physical performance.

## Figures and Tables

**Table 1:** Baseline mean (SD) characteristics for bone and physical fitness and adjusted mean difference in percent (95% CI) between fallers without (Nonfracture) and with fractures (fracture).

Baseline	Nonfracture *N* = 120	Fracture *N* = 67		*P* (Anova)
Age (years)	62.5 (5.4)	63.3 (5.4)		0.35
Height (cm)	161.1 (5.7)	160.9 (5.3)		0.79
Weight (kg)	68.1 (10.3)	66.4 (9.4)		0.26
DXA			Adjusted mean difference^1^	*P* (Ancova)^1^
Femoral neck BMD (g/cm^2^)	0.823 (0.129)	0.776 (0.098)	4.5 (0.3 to 8.6)	0.035
Trochanter BMD (g/cm^2^)	0.907 (0.129)	0.859 (0.098)	4.3 (0.5 to 8.0)	0.028
Distal radius BMD (g/cm^2^)	0.358 (0.059)	0.316 (0.053)	11.1 (6.3 to 16.1)	<0.001
pQCT, distal tibia				
Bone mineral content, BMC (g)	647.9 (84.4)	604.8 (83.1)	6.0 (2.2 to 9.7)	0.002
Trabecular density (g/cm^3^)	224.1 (28.6)	214.0 (33.3)	4.1 (0.1 to 8.2)	0.053
Section modulus (mm^3^)	855.2 (287.0)	752.4 (250.0)	9.9 (0.1 to 19.7)	0.049
pQCT, tibial shaft				
Bone mineral content, BMC (g)	768.9 (86.6)	735.02 (86.8)	3.6 (0.4 to 6.8)	0.027
Cortical density (g/cm^3^)	1097.7 (32.2)	1095.2 (30.1)	0.2 (−0.6 to 1.0)	0.64
Cortical area (mm^2^)	280.0 (28.7)	268.3 (29.3)	3.4 (0.6 to 6.2)	0.019
Section modulus (mm^3^)	1658.3 (211.4)	1606.6 (242.2)	2.3 (−1.4 to 6.1)	0.22
Physical performance				
Figure-8 running (s)	17.7 (2.1)	18.0 (2.1)	−1.9 (−4.9 to 1.1)	0.20
Jumping test (cm)	18.7 (4.1)	18.3 (4.0)	2.6 (−3.4 to 8.7)	0.40
Grip strength, right hand (kg)	30.5 (4.8)	29.2 (4.1)	0.1 (0.0 to 0.2)	0.14
Isometric leg-extensor strength (N/kg)	18.3 (3.4)	17.9 (3.5)	2.5 (−2.3 to 7.2)	0.31
VO_2max_ (ml/min/kg)	28.2 (5.7)	28.6 (5.8)	−0.6 (−4.7 to 3.4)	0.77

^1^Adjusted for age, height, and weight.

**Table 2:** Baseline characteristics in control and exercise groups divided into fallers without fracture (nonfracture) or fallers with fracture (fracture) and adjusted mean difference (95% confidence intervals).

	Controls, *N* = 84		*P* (Anova)	Exercisers, *N* = 103		*P* (Anova)
	Nonfracture *N* = 55	Fracture *N* = 29		Nonfracture *N* = 65	Fracture *N* = 38	
Age (y)	61.7 (4.8)	62.9 (4.1)	0.25	63.1 (5.8)	63.5 (6.2)	0.66
Height (cm)	161.2 (6.0)	161.8 (5.6)	0.66	161.1 (5.4)	160.3 (5.0)	0.25
Weight (kg)	69.4 (10.9)	66.7 (9.4)	0.25	67.0 (9.7)	66.1 (9.5)	0.83
DXA			Mean difference^1^			Mean difference^1^
Femoral neck BMD	0.824 (0.122)	0.764 (0.107)	4.8 (−1.3 to 10.5)	0.822 (0.135)	0.785 (0.091)	3.8 (−1.9 to 9.6)
Trochanter BMD	0.906 (0.116)	0.840 (0.106)	5.4 (−0.1 to 10.7)	0.909 (0.140)	0.874 (0.090)	3.4 (−0.2 to 9.2)
Distal radius BMD	0.367 (0.063)	0.314 0(.053)	13.4 (6.4 to 22.3)^2^	0.350 (0.055)	0.318 (0.054)	9.4 (3.1 to 15.7)^2^
Distal tibia, pQCT						
BMC	636.4 (86.3)	581.2 (84.0)	7.7 (1.6 to 13.8)^2^	657.7 (82.1)	622.8 (78.7)	4.8 (0.2 to 9.5)^2^
TrD	222.4 (30.0)	208.8 (35.8)	4.8 (−2.2 to 11.8)	225.6 (27.5)	217.9 (31.2)	3.9 (−1.2 to 9.0)
BSI	864.9 (292.4)	725.3 (252.2)	10.1 (−4.6 to 24.9)	847.0 (284.4)	773.2 (249.7)	8.1 (−5.2 to 21.5)
Tibial shaft, pQCT						
BMC	762.8 (84.3)	727.0 (95.3)	3.6 (−1.3 to 8.5)	774.1 (88.8)	741.5 (80.5)	3.6 (−0.6 to 7.8)
CoD	1100.3 (30.5)	1096.2 (28.4)	0.3 (−0.9 to 1.6)	1095.5 (33.7)	1094.5 (31.7)	0.1 (−1.1 to 1.2)
CoA	277.0 (27.6)	265.1 (33.1)	3.2 (−1.1 to 7.5)	282.5 (29.5)	270.7 (26.1)	3.5 (−0.1 to 7.2)
BSI	1629.0 (197.6)	1615.1 (251.5)	0.3 (−5.4 to 6.0)	1683.0 (220.9)	1600.1 (238.0)	4.2 (−0.9 to 9.2)
Physical performance						
Figure-8 run (s)	18.5 (2.4)	18.4 (2.3)	−0.7 (−6.0 to 4.3)	17.1 (1.7)	17.7 (1.9)	−3.1 (−6.0 to −0.6)^2^
Jumping height (cm)	17.3 (3.4)	18.3 (3.8)	−3.8 (−12.0 to 4.4)	19.9 (4.3)	18.4 (4.2)	7.6 (−0.8 to 15.9)
Grip strength, right hand	30.2 (4.5)	30.0 (4.3)	2.3 (−5.0 to 9.7)	30.8 (5.1)	28.6 (4.0)	4.5 (−1.1 to 10.1)
Isometric leg extensor (N/kg)	17.2 (2.9)	17.7 (2.8)	−2.8 (−9.5 to 4.0)	19.2 (3.5)	18.1 (3.9)	6.4 (−0.6 to 13.3)
VO_2max_ (ml/min/kg)	26.5 (4.9)	27.4 (5.6)	−0.9 (−7.2 to 5.4)	29.6 (6.1)	29.5 (5.8)	−0.3 (−5.1 to 4.5)

^1^Adjusted for age, height, and weight. ^2^*P* < 0.05.

## Data Availability

The datasets analyzed during the current study are not publicly available because only limited access to the use of the patient-register data was approved by the Department of Social Services and Health Care of the City of Tampere but are available from the corresponding author (KU-R) on reasonable request.
